# Perceptions about trust: a phenomenographic study of clinical supervisors in occupational therapy

**DOI:** 10.1186/s12909-019-1850-1

**Published:** 2019-11-04

**Authors:** Pernilla Lundh, Per J. Palmgren, Terese Stenfors

**Affiliations:** 1 Karolinska University Hospital, Stockholm, Sweden; 2 Department of Neurobiology, Care sciences and Society, Karolinska Institutet, 141 86 Huddinge, Stockholm, Sweden; 3 Department of Learning, Informatics, Management and Ethics, Karolinska Institutet, 171 77 Stockholm, Sweden

**Keywords:** Continuing education, Education, Learning, Occupational therapist, Phenomenography, Qualitative research, Trust

## Abstract

**Background:**

Finding the best way to facilitate student learning in clinical practice can be challenging for clinical supervisors. While high levels of trust might jeopardize patient safety, low trust might hinder student learning; however, carrying out professional activities is necessary for students to develop professional competence. There is a dearth of scholarly literature regarding the concept of trust among clinical supervisors in occupational therapy education. A better understanding of how trust is created between the supervisor and student may thus aid in facilitating student learning. The aim of this study, therefore, was to explore occupational therapy clinical supervisors’ perception of trust and how it is formed.

**Methods:**

A qualitative method deploying a phenomenographic approach was chosen. Twelve clinical supervisors were interviewed, and the data were analyzed according to the seven-step phenomenographic approach.

**Results:**

Three qualitatively different ways of thinking about trust were found: (1) that trust is about the student and is rather static; (2) trust as a dynamic process based on student performance; and (3) trust as something mutual and interrelated. The findings indicate that trust can be understood in various ways, such as being something inherent in the student or, alternatively, about the student, the supervisor, the relationship between them, and the surrounding context, including the tasks performed. Furthermore, the study shows that trust can be seen either as something static or as a dynamic process.

**Conclusions:**

This study contributes to a deeper understanding of the variation of ways in which the concept of trust is understood among clinical supervisors in occupational therapy. The study corroborates the prior research finding that trust can be understood as a multifaceted construct. It contributes novel insights about the role of the supervisor as an influential factor in the trust-building process. A deep understanding of the possible differences in the ways of conceptualizing something can help supervisors support learning by building on this understanding. The results from this study contribute to our knowledge of the drivers behind entrusted decisions in clinical education in various professional contexts. We suggest that the results be used in the continuing professional development of clinical supervisors.

## Background

In the sphere of health care, the clinical environment is innately complex and venturesome. In healthcare professions education, clinical placements are considered indispensable to all healthcare students due to the noteworthy emphasis on the learning of skills and attitudes, which cannot be acquired in the absence of experience in a clinical setting. However, organizing clinical supervision in a way that sufficiently takes into account student learning and patient safety can be challenging. The patient trusts the clinical supervisor and expects high quality care and optimum standards of safety. Likewise, the clinical supervisor needs to trust that the student is doing the right thing in the right way and at the right time.

Scholars have highlighted that in healthcare professions education, the very use of the word “entrustment” conveys new attention to the notion of trust [1]. Thus, any engagement with the concept elicits an array of questions. While the importance of the concept of trust is probably undisputed, there is a lack of a coherent definition, and a common demarcation of its components remains elusive. One definition was recently advanced by Damodaran et al. [1]: “Trust is a judgement by the trustor, requiring the acceptance of resultant vulnerability and risk, that the trustee (individual or organization) has the competence, willingness, integrity and capacity (i.e. trustworthiness) to perform a specified task under particular conditions.”

It has been argued that the idea of trust reflects a dimension of competence that extends beyond observed ability [2]. Thus, entrusting a professional activity should lead to the trainee being granted responsibility in all similar future circumstances. One of the roles of the supervisor is to challenge students by providing them with appropriate professional activities that align with their current level of competence [3, 4]. The scholarly literature has identified that high levels of trust in a student might jeopardize patient safety and that low trust might hinder student learning [5]. From a student perspective, early engagement in patient care can facilitate feelings of authenticity [6] and can be of relevance to student learning [7, 8]. Carrying out professional activities is necessary for students to develop professional competence and, thus, become better prepared for their future professional role [6, 9]. However, despite knowledge about the importance of providing enough of a challenge for students to grow as well as providing them with real patient encounters, there is considerable variation among clinical supervisors regarding the reasons and timing for allowing students to perform various patient interventions [4]. As a result, students’ learning experiences from clinical education might vary considerably [3, 4, 10], with the consequence of some students being poorly prepared for the complex reality as professionals and potentially jeopardizing patient safety.

Hauer et al. [5] have suggested that trust in clinical supervision is influenced by five factors: the supervisor, the student, the relationship between the supervisor and the student, the context, and the task. The supervisor’s contribution to trust is affected by their own clinical and supervision expertise [5] as well as by their attitudes, habits, and responsibility toward both the student and patient [5, 11, 12]. Competence, attitude, habits, and self-confidence have been suggested as some of the contributions by students in establishing trust [5]. The relationship between the student and supervisor [5, 13] as well as between the context and task [4, 5] has also been seen as influential in relation to trust. Workplace affordances, work environment, system issues, workload, and workplace culture have been identified [5] as contextual factors that can variously impact on trust. Moreover, a more deliberate way of creating learning tasks for students, i.e., by means of sequencing, has been advocated as a means to ensure both student learning and patient safety [14].

Multiple theoretical perspectives can inform a deeper understanding of how trust impacts clinical supervision. However, given the relevance of contextual and socio-cultural aspects of the triadic relationships among the supervisor, the student, and the social context in which learning occurs, a socio-cultural perspective can illuminate clinical supervisors’ experiences of trust within a social context [15].

Informed by research and theory, we identified a gap in the literature linked to how clinical supervisors understand the meaning of trust as they supervise within the workplace. Empirical investigations on trust have been conducted in clinical supervision within several healthcare professions, such as medicine, nursing, and psychology [4, 5, 7, 11, 13, 14, 16–23]. However, to date, no research has been identified regarding how clinical supervisors in occupational therapy education perceive trust, thus providing us with a motivation to investigate this cohort.

We posed the following overarching research question: “How can student learning be supported through a better understanding of the manner in which trust is created between the supervisor and student?” A deeper understanding of the meaning of trust can stimulate reflection and help facilitate the development of learning environments that are apt for healthcare professional students. Thus, the aim of this study was to explore occupational therapy clinical supervisors’ perceptions of trust and how it is formed.

## Methods

### Design and theoretical assumptions

To ensure congruency with the aim of the study, a qualitative method employing a phenomenographic approach was chosen. Phenomenography (not to be confused with phenomenology) is the empirical study of variation in the ways in which people understand or experience phenomena in the world around them and how these ways of understanding are logically and hierarchically related to each other and to the perceptions of the situations in which they are experienced. The focus on variation is one of the strengths of the phenomenographic approach, which has been extensively described in the literature [24–27]. In phenomenography, learning is designated as a change in the internal relationship between the world and the individual [28], suggesting that it concerns our experiences or the way in which something is perceived [24, 28]. Phenomenography is based on a non-dualistic ontology, which means that the world is accessed through our experience of it. Accordingly, Marton and Booth [28] have postulated that the description of the world, the describer, and that which is described cannot be separated from each other. Epistemologically, the assumption of phenomenography is that individuals differ in terms of how we experience the surrounding world, although the differences can be described, related to, and understood by others. The conceptions may vary from one individual to another and within the same person, as different aspects of the phenomenon are conceived according to its wholeness in relation to a given context [24, 28].

### Participants and setting

The study was conducted at a large emergency university hospital in Sweden, where all occupational therapists at the clinic are expected to supervise students. At the time of the study, there were students from semesters 1, 2 (placements lasted 2 weeks/semester), 3 (3 days of placement), 4 (placement lasted 11 weeks), and 5 (placement lasted 5 weeks). Semester 6 students attended an interprofessional educational ward for two weeks and sometimes spent two additional weeks undertaking an advanced clinical elective. There were also students from other countries for different periods of time as well as two mentors who supported the supervisors and students. The mentors were occupational therapists who had long experience as clinicians and as supervisors for undergraduate students. There were two adjunct clinical lecturers who functioned as a link between the university and the university hospital. They were responsible for continuous education in clinical supervision and pedagogical support of the supervisors.

In occupational therapy in Sweden, it is a common practice for supervision to be set up as a one-to-one relationship between the supervisor and student. Sometimes, there are other constellations, such as two supervisors and one or two students. Occupational therapists work on a consultancy basis at the hospital and visit different wards. More complex professional activities within occupational therapy can consist of a personal care assessment at the ward with a patient who suffers from stroke and struggles with poor balance and diminished cognitive capacity. Occupational therapists sometimes work in interprofessional teams consisting of nurses, physiotherapists, assistant nurses, physicians, speech and language therapists, dieticians, among others. Some of them are partly or fully stationed at different outpatient clinics.

A purposeful maximum variation sampling strategy was used to obtain breadth in the data [29, 30]. Thus, all the occupational therapists with clinical supervisor experience at one hospital (*n* = 65) were invited to participate. Twelve occupational therapists, representing five out of six occupational therapy offices in the hospital, volunteered to participate in the study. The characteristics of the participants are presented in Table 1. The clinical work experience among the participants included patients of all ages, from children to the elderly, with variations in diagnosis and type of care, such as inpatient and outpatient clinics. Three-fourths of the participants had undergone supervision training.

**Table 1:** Participant characteristic (*n* = 12)

Gender	9 females
	3 males
Range of clinical experience	6 months – 40 years (median 15 years)
Range of supervisor experience	3 months – 30 years (median 8.5 years)
Orientation of work	Neurology, Rheumatology, Orthopedics, Burn Injury, Gastroenterology, Pediatrics, Geriatrics, Memory Investigations, Neuropediatrics, Acquired Brain Damage, Clinical Educational Ward, Infectious Diseases and Inflammation, Emergency Unit
Supervisor training	9 of 12

### Data collection

The use of semi-structured interviews is the most common method of collecting data in phenomenography and was the approach adopted in the present study. The first author (PL), who has had previous experience of conducting qualitative interviews, carried out the interviews. The interviews were conducted at a location of the respondents’ choosing and lasted between 28 and 56 min. The interviews were audio-recorded and subsequently transcribed verbatim, yielding more than 8 h of recorded material and resulting in 179 pages of textual data. After the first interview, the first and last authors read the transcript and made marginal modifications to the interview guide. This process was repeated after the second and third interviews. Appropriate follow-up questions in the interviews were facilitated by the fact that the interviewer was of the same professional background as the study participants and had several years of experience as a clinical supervisor.

### Data analysis

Soon after the first interview, the data analysis began by means of keeping a diary of immediate thoughts about what the interviewee was “talking” about. During the process of transcribing the interviews, this diary was also used to collect immediate thoughts. The transcribed interviews were read and analyzed by the first (PL) and last (TS) authors, both separately and together. The analysis of the interview data followed the seven steps described by Dahlgren and Fallsberg [31], presented in Table 2.

**Table 2:** The seven steps of phenomenographic analysis

1. Familiarization	Reading through all interview transcripts in depth to get an impression of how the interview proceeded. All data in the entire pool are given equal consideration.
2. Condensation	Identifying meaning units in the dialogue of each interview and marking or saving these for further scrutiny.
3. Comparison	Comparing each of the meaning units for similarities and differences.
4. Grouping	Allocating answers expressing similar ways of understanding the phenomenon to the same category.
5. Articulating	Capturing the essential meaning of a certain category.
6. Labelling	Expressing the core meaning of each of the categories. Steps 3–6 are repeated in an iterative procedure to make sure that the similarities within and differences between categories are established.
7. Contrasting	Comparing the categories through a contrastive procedure whereby they are described in terms of their individual meanings as well as in terms of what they do not comprise.

Although these steps seem sequentially ordered, the analytical process was not linear; rather, there was a constant interplay between the steps. More specifically, the analysis began following the completion of the transcription process, with PL reading through all the transcripts to get a feeling of the participants’ perceptions regarding trust. As a way of becoming familiar with the data at the beginning of the analysis, four questions were posed in relation to the text: *What differences can be found in the ways of understanding trust? What differences can be found regarding how to develop a trustful relationship between the supervisor and student? What factors can be found to explain differences in thinking about trust and the trust-building process? What are the consequences of a high level of trust and a low level of trust?* In employing an inductive approach to the analytic process, the researchers endeavored to keep an open mind about the possible findings in the text itself. The NVivo (2.0.) software was used to group and articulate the data. Through an iterative process of repeatedly moving between the analysis and reading the transcripts, the categories were labelled and contrasted, resulting in distinct, qualitatively different categories. As part of this process, the relationships between the categories were scrutinized, resulting in a hierarchy between the categories, with the first representing a more limited understanding of the phenomenon than the latter, which included additional aspects and dimensions of trust [24]. The findings were discussed and subjected to adjustments until a consensus among the investigators (PL and TS) was reached. Frequent debriefing sessions among all the investigators ensued throughout the process.

We took steps to enhance the trustworthiness of the findings. We used reflexivity as a technique for establishing confirmability [32] by including a clinician who served as a supervisor, and who experienced trust in learners, as well as non-clinician researchers who, throughout the analysis, jointly engaged in a discourse to scientifically query and challenge one another’s assumptions. The research team also used a process of investigator triangulation [25]. Two investigators (PJP and TS), who were not directly involved in the coding, reviewed the major findings and used their expertise in supervision, medical education, and research to help clarify and critique the findings.

## Results

This paper draws on perceptions of the meaning of trust among twelve clinical supervisors in undergraduate healthcare professional education. The analysis resulted in three different categories of the concept of trust: that trust is about the student and is rather static; trust as a dynamic process based on student performance; and trust as something mutual and interrelated. Each category is illustrated with supporting quotes.

### That trust is about the student and is rather static

In the first category, trust was understood as a feeling that was rather static, formed on the basis of demographic factors in relation to the student, such as educational background and gender, as well as from the first impression of the student’s personality, like-mindedness regarding learning style, and ways of socializing. Gender was considered by a few participants to influence trust in their students. A feeling of belongingness with students of the same gender or, alternatively, difficulty interpreting students of the opposite gender from oneself were given as reasons for facilitating, hindering, or prolonging the trust-building process.

… I immediately felt a sense of belonging because he was a man (yes), and it is not so common that it is a man in this profession (no), so it became … one can say that it was a little unfair towards the other [a female student], since he immediately got some benefits… (Participant 8)

No, I don’t think so […] not here … but it is something one should consider, so ehm … of course because male students are rare here to us, and I might feel that it is easier to… I could have that bias that I can more easily interpret female… (Participant 3)

Students’ personality traits, such as being open, active, and eager, were considered by many participants to facilitate trust. Being brave and confident and taking initiative were expressed by some as important personality traits among students. Being quiet, cautious, or passive as a student were seen by some as personality traits that prolonged the trust-building process. Like-mindedness regarding learning-style preferences, such as wanting to discuss, daring to challenge one’s discussion partner, and being able to argue in support of one’s case were expressed by a few as factors that generate trust. Some maintained that being openly curious influenced trust in a positive way.

Curious, maybe, that’s it, I think, asks a lot of questions (mm) because those [students] you have felt that you cannot fully leave, they haven’t asked much (mm) … even though you say in the beginning that it isn’t a sign of weakness [to ask], rather the opposite. I think that many [students] think that “well then, she thinks that I don’t know…” and it is quite the opposite. I think that those who are curious are those who ask a lot and want to participate, and, well, yes, I think that is actually what it is all about… (Participant 7)

### Trust as a dynamic process based on student performance

In this category, trust can be described as a feeling that grows stronger or weaker depending on the student’s performance in various professional activities. In this dynamic process of trust building, the supervisor looks for signs of maturity and readiness in the student before allowing the student to advance to the next level or milestone. Professional conduct and good communication were seen by some to be important signs of readiness among students.

…I dared to let her go fairly soon because I knew that she wouldn’t do an assessment or intervention until she had spoken with us and that we would get a chance to assess the appropriateness of her conclusion (mm) eh so that … well that was it and also that I knew that she cared about the patients and that she remained within the boundaries that she was supposed to when talking to the patients eh … none of her own opinions about politics at large … or how the system works on a societal level… (Participant 6)

Some participants thought about how the students sought and responded to feedback. Showing interest in occupational therapy, demonstrating a willingness to participate, posing relevant questions, and being transparent about their own clinical reasoning were other signs that many supervisors were looking for in students.

To trust (yes) eh … firstly, I want to know that the student has gained the knowledge that she needs to be able to proceed (mm), and that is what you do in dialogue with the student and feedback all the time. That’s when I feel that she has reached that level of understanding of what we do as occupational therapists, our occupational therapy process, and understands the essence. This is where I understand that she has understood the underlying theories and then the next step is that the competence should be there, where she dares to do it, that she feels safe in doing it… I don’t want to force a failure; so instead, I give space for them to reach that thought themselves. (Participant 4)

### Trust as something mutual and interrelated

In the final category, trust was characterized as something that was cultivated equally between the supervisor and student. Here, trust was described as a feeling that became stronger or weaker the more the student and supervisor got to know each other. Trust was seen as being formed not only by demographic factors and the personality traits of the student, or the dynamic process based on student performance, but also by mutuality and interrelatedness. Mutuality is about giving and taking from both parts, whereby the student has a chance to learn about the supervisor and vice versa. The student and supervisor were both seen as learners.

Eh, I think it is there in the space between the supervisor and student…. I think it is that magical thing that happens in our communication when we meet, and sometimes, one has to work a little harder to get this eh … well when it says click eh so … where you actually understand each other so (mm), and hopefully, you reach that during these weeks. (Participant 3)

Issues relating to language and communication and having a dialogue between the supervisor and student—characterized by mutual openness, honesty, bravery, and humor—were also described as facilitating trust. Lack of an open dialogue was seen to influence trust in a negative way. An open dialogue was thought to facilitate the process of finding a student’s current and true level of competence.

Lack of trust can occur like, “well why didn’t they say so in the first place!” But sometimes, it can be that they [the students] sometimes think that they can do more than they actually can, and it is not until they are faced with these situations that it is revealed. It can be something latent, and they think that it will work … and they rarely want to say no because it is so important with the practical work, but when you “show all the cards,” that’s when it turns out great in the end. (Participant 2)

An open dialogue was also seen to facilitate shared mental models about learning styles or intended learning outcomes and how to get there together as a team. In this category, trust was seen as a possible achievement, regardless of the student’s previous achievements at other placements. Trust was understood not only in terms of trusting the student but also trusting oneself as an occupational therapist and supervisor. Some supervisors did not only expect the student to change; they were also ready to change themselves and, hence, influence the trust-building process by adopting new ways of supervising. Being trusted as a supervisor was not taken for granted by some of the participants, who felt that they had to earn trust by making students feel safe. Others expressed this in the following way: “by giving trust, you receive trust.” Some participants expressed that they were often open toward students in a purposeful way, thereby exposing their own vulnerability and imperfection and hoping that this would make the student feel safer and braver about asking questions.

…I say [to the students] “Take the grain of gold [best qualities] from us all” … eh… “we are not perfect, none of us.” One of us documents in the patient’s journal very well; someone else is good at transfers; and the third person is excellent at making external orthoses. Take the grain of gold from us all, and you will have the best. Because nobody is perfect, and if you dare to show that you are not perfect but you are good at many things, then perhaps you can actually show a future colleague, a student, that it is ok to fail. You may ask your colleagues, even if you have 20 years of experience, “what would you do in this case?”…that you dare to show to the students that it is about never-ending learning. It never ends. (Participant 2)

Student participation was brought up by several participants, some of whom worked purposefully to allow students to take part in patient encounters as early and as frequently as possible. Making the student feel like part of the work group in the Occupational Therapy Department was another aspect of participation that was considered by some to facilitate the trust-building process. Some supervisors also expressed the importance of being mindful not to develop an overly close personal relationship with the student and risk losing the ability to make unbiased and well-informed decisions about student competence and patient safety.

This category was also about interrelatedness, encompassing other aspects of the learning environment besides the student, the supervisor, and their relationship:

Without the patient, I wouldn’t think about trust. (Participant 4)

Trust was described as finding the perfect match between student learning, patient safety, and the supervisor feeling safe. It was about communication between several parties, such as the student, the supervisor, and the patient and their next of kin. The level of independence was considered by some to be affected by the context, such as other staff members, patients, or the design of the localities, and by the complexity of the professional task, such as the status of the patient, the risk of injury, and technical aspects.

## Discussion

Outcomes in phenomenography focus on variation in the experiences regarding a given phenomenon and how these variations are related to each other [25]. The findings of this study present variations in how clinical supervisors think about trust in the context of their supervision. These variations ranged from a more limited perspective of trust as a feeling within the clinical supervisor—which could be static or dynamic in nature, and based on demographic facts, personality traits, and behavior—to aspects such as the relationship between the student and supervisor, other perspectives such as the learning environment and the task at hand, and trust as a process that can be actively influenced through supervision. The consequences of trust were related to the ways in which the supervisors thought about independent practice and learner autonomy as well as about the context and tasks.

Some clinical supervisors perceived trust in relation to the student in a rather static way. The expression “rather static” implies that trust is formed based on a way of thinking of humans, in general, that is not so easily altered. Learning in a clinical context is experiential, situated in communities of practice and an environment in which real work is ongoing. Students start off in legitimate peripheral participation [33], progressively increasing their understanding and engagement and, over time, becoming increasingly central to the decision-making process. Thus, the rate of this approximation might be influenced by personal traits and characteristics. For instance, student personality traits, such as being open and having a willingness to discuss, were seen by some as signs of a trustworthy person, whereas quiet and cautious students were considered more difficult to trust. Furthermore, same gender was thought of by some to facilitate communication and the “getting to know you” process, thus speeding up the trust-building process. Ginsburg et al. [12] found that student personality traits affected the evaluator’s opinions of the student, although personality traits were not necessarily related to trust.

Our findings in the second category show that trust was perceived in the context of the student being more dynamic in nature, thus being influenced by performance. These findings resonate with the prior research emphasis on the agency of the learner [5, 20, 34]. The development of trust can thus be facilitated through observation of the student and their actions. Students’ attitudes and habits of mind have been highlighted as influential factors in the model of trust presented by Hauer et al. [5], as these factors can reveal how students seek and respond to feedback, which in turn can influence supervisors’ trust in their students. Bowen [16] found that when medical students were transparent about their diagnostic reasoning skills, supervisors could more confidently entrust them with less supervised practice. Other signs of readiness for independent practice delineated by the participants in our study included when students were self-reflective about their own strengths and weaknesses, sought and responded effectively to feedback, and were able to handle critical situations. These results find resonance in prior research in which self-awareness and life-long learning skills have been seen as important student qualities for entrustment [5, 22, 35, 36]. Coping with mistakes and demonstrating safe clinical practice and risk management behavior were found to influence physician clinical educators’ trust in their trainees [22].

In the categories in which trust was experienced *as a dynamic process based on student performance* and *as something mutual and interrelated*, trust was linked to both student and supervisor behavior. While there are many other trust relationships in healthcare professional education, Damodaran et al. [1] have asserted that at the most basic level, it is helpful to clearly delineate who are the trustor and trustee and what task they perform. Student behavior—in terms of professional conduct, communication, seeking and responding to feedback, willingness to participate, posing relevant questions, and being transparent about their own clinical reasoning—was considered by the respondents to influence the trust-building process between the supervisor and student. Many participants also linked trust to making the student feel safe and described various ways of creating a safe learning environment. This could take different forms: letting students share the same office as the rest of the work group, including students in lunch breaks, using humor to try to make them feel good, or showing one’s own vulnerability as a supervisor, not being perfect, though good at many things. It could also encompass letting students assume partial responsibility for taking progressive steps in their own learning process, which could mean encouraging them to think out loud in terms of their readiness for meeting a certain patient with a specific problem. These findings resonate with prior research. Costa et al. [11] have argued that behaviors of trust can be seen as a component of trust and can be of either a cooperative or monitoring nature. Cooperative behaviors refer to open communication about team members’ work, feeling personally involved with the team, and accepting the influence of others. Conversely, monitoring behaviors come into play in the absence of trust and align with a necessity to control other members’ work and exercise surveillance [11]. Hauer et al. [18] found that supervisors differed in their characterization of the meaning of trust based on how much time they spent checking residents’ work. They also found that a supervisor’s style, i.e., their propensity to trust less and check residents’ work more, could be perceived by physician clinical supervisors as a barrier to trust formation [18].

The findings of our study indicate that trust can sometimes be perceived as processual. Thinking about trust as a dynamic process makes way for thinking about how trust changes over time. Some participants expected braveness, the ability to take initiative, and the ability to question supervisors’ choices to feature among students from the very beginning of the practice placement period, whilst others expected to see these behaviors later on. Other researchers have found that at the beginning of the practical placement, the supervisor’s trust in the student can be based on the latter’s demographic background and that, later on, it can be based more on mutual dialogue about, for instance, how to solve problems [13].

The findings of this study lead to further reflections about the level of independence students can achieve during clinical practice versus learner autonomy. The study participants talked about student independence in various ways, from being dependent on the supervisor to being independent without the supervisor in the room, though easily available if needed. Factors such as the age of the patient, the type of care (outpatient clinic), and student characteristics were discussed as influential in full entrustment. According to some, full entrustment regarding unsupervised professional activities may never happen in undergraduate medical education for most tasks [14]. Nevertheless, a gradation of the supervision of entrusted professional activities promotes progressive learner autonomy [14]. Being an autonomous learner does not necessarily mean being independent; rather, it means being in charge of your own learning process [36].

A limitation of this study is that maximal variation might not have been fully attained, as all the respondents worked at the same hospital, albeit in five different offices. However, their variation in experience must be regarded as strength of the study. Moreover, the number of interviews is in line with the phenomenographic approach employed. The description of the contextual setting, the participants, and the analysis, together with the links drawn between the findings, the theory, and the prevailing literature, may create possibilities for the reader to appraise the transferability of the results. Moreover, the serene and open interview situation promoted an extensive dataset for the analysis. The richness of the data, together with frequent debriefing sessions and investigator triangulation enhanced the credibility of the results [29, 37].

In line with the phenomenographic approach, the categories presented in the findings do not necessarily represent specific respondents, as a descriptive collection of such unique conceptions is considered less useful when it comes to guiding, for example, educational change than the related categories that a phenomenographic analysis provides. Instead, the focus is on qualitative differences and critical variations in the ways of understanding the phenomenon at hand. To train future colleagues to be as ready as possible for independence and patient safety practices, clinical supervisors may wish to reflect on their responsibility in the trust-building process. Moving beyond first impressions of students based on demographic factors and student performance toward a more mutual process of getting to know each other in terms of expectations, ways of thinking about humans in general, individual learning styles, and level of competence, including one’s own vulnerabilities, seems to have a positive impact on the trust-building process from a clinical supervisor point of view. Our findings suggest that trust can be seen as a learning process in which both the student and supervisor are active learners. Trust development seems to be facilitated by an awareness—among all actors in the pedagogical encounter—of mutuality as a prerequisite for successful trust building and potential interrelatedness. The findings of this study can be used as a starting point to reflect on these issues in, for example, supervision training. The findings are potentially transferable to other professional contexts within healthcare, as all professions share the aim of providing high quality care, where patient safety must be assured even during the training of future professionals.

## Conclusions

This study contributes to a deeper understanding of the variation of ways in which the concept of trust is understood among clinical supervisors in occupational therapy. The findings indicate that trust can be understood in various ways, for example, as something inherent in the student or, alternatively, about the student, the supervisor, the relationship between them, the surrounding context, and the tasks performed. Furthermore, the study shows that trust can be seen either as a static or dynamic process. This study corroborates prior research that trust can be understood as a multifaceted construct and contributes with further insights about the role of the supervisor as an influential factor in the trust-building process. A deep understanding of possible differences in the ways of conceptualizing something can help supervisors support learning by building on this understanding.

## Data Availability

The datasets used and/or analyzed during the current study are available from the first author on reasonable request.
